# Outcomes of Ewing sarcoma in adults over 40 years of age from a low-middle income country

**DOI:** 10.3332/ecancer.2022.1361

**Published:** 2022-03-01

**Authors:** Goutam Panda, Arun Chandrasekharan, Shasanka Das, Prabhat Bhargava, Sujay Srinivas, Siddhartha Laskar, Smruti Mokal, Bharat Rekhi, Nehal Khanna, Nandini Menon, Vijay Patil, Vanita Noronha, Amit Joshi, Kumar Prabhash, Shripad D Banavali, Sudeep Gupta, Jyoti Bajpai

**Affiliations:** 1 Department of Medical Oncology, Tata Memorial Hospital, Homi Bhabha National Institute, Mumbai, 400012, India; 2 Department of Radiation Oncology, Tata Memorial Hospital, Homi Bhabha National Institute, Mumbai, 400012, India; 3 Department of Biostatistics, Tata Memorial Hospital, Homi Bhabha National Institute, Mumbai, 400012, India; 4 Department of Pathology, Tata Memorial Hospital, Homi Bhabha National Institute, Mumbai, 400012, India

**Keywords:** Ewing sarcoma, ≥40 years, older adults, survival, chemotherapy-toxicity

## Abstract

**Introduction:**

The data on outcomes and toxicity in adult Ewing sarcoma (ES) patients, particularly those aged ≥40 years, is exceedingly scarce around the world, particularly in low- and middle-income countries (LMICs) and mandates research.

**Methods:**

The study involved histologically ascertained ES patients aged ≥40 years who registered at our institute from 2013 to 2018. Prospectively collected data were analysed for overall survival (OS), event-free survival (EFS) and chemotherapy-related toxicities.

**Results:**

There were 66 patients, of which 34 were non-metastatic, and 32 were denovo metastatic, recurrent or had doubtful metastasis. At presentation, median age was 46 years, and 42 (63.6%) had extra-skeletal primary and 24 (36.3%) had extremity tumours. Curative treatment was offered to 40 (60.6%) patients. Significant grade 3/4 toxicities in non-metastatic and metastatic cohort, respectively, were febrile neutropenia (61.3%, 37.5%), anaemia (58.1%, 37.5%), thrombocytopenia (45.2%, 25.0%), peripheral neuropathy (25.8%, 12.5%) and dyselectrolytemia (25.8%, 6.25%). Chemotherapy-related toxicity led to death in three patients in the metastatic cohort, versus none in the non-metastatic patients. The 5 year EFS and OS for non-metastatic cohort were 53.8% and 67.8%, while the same for metastatic cohort were 20.7% and 27.5%, respectively. On multivariate analysis, Eastern Cooperative Oncology Group-performance status >2 and metastasis at presentation predicted poorer EFS and OS. Additionally, raised lactate dehydrogenase, larger tumours (>8 cm) and palliative intent treatment predicted worse EFS, while extra-skeletal primary and female gender were indicators of worse OS.

**Conclusions:**

Older adult ES patients benefit from aggressive multimodality treatment even in LMIC infrastructure. However, careful patient selection, close monitoring and pertinent dose modifications is imperative due to higher propensity for potential toxicities.

## Introduction

Ewing sarcoma (ES), an uncommon and aggressive cancer, is predominantly seen in children and young adults [1]. It belongs to the Ewing family of malignancies, the others being primitive neuroectodermal tumour and Askin’s tumour [2, 3]. Systemic spread is a hallmark of ES, and thus employs the early use of chemotherapy (including doxorubicin, cyclophosphamide, vincristine, dactinomycin, ifosfamide and etoposide) followed by amputative/limb-sparing surgery or definitive chemo-radiation. This multi-disciplinary treatment has brought about considerable betterment in prognosis, from under 10% to 70%–80% [4–12]. Recent studies have shown that dose-dense chemotherapy leads to improved outcomes and is considered as the current standard treatment. However, this information comes from randomised studies conducted in children with a meagre representation of the adult population. At diagnosis, the median age for ES patients is 15 years and ES is far less common over 40 years [13]. Adults form a challenging subgroup in view of multiple comorbidities like impaired renal function, hepatic disease, decreased bone marrow reserve, cardiac issues disease and polypharmacy. This makes them more susceptible to chemotherapeutic agents’ various toxicities and unlikely to tolerate the dose-dense, standard chemotherapy regimen. There is a dearth of data with respect to survival and chemotherapy toxicity of older ES patients over 40 years, as most studies exclude this cohort. Historically, it has been observed that older ES patients performed poorly compared to children [14, 15], and a new French study supported this view as well [16]. Notwithstanding, some authors have reported both groups having comparable results at the cost of increased chemo-related toxicity in older patients [17, 18]. Given this conflicting data, we undertook a study to analyse the survival, toxicity and factors affecting outcomes in the older ES patients over 40 years registered at our centre.

## Methods

The data were obtained prospectively from histologically ascertained ES patients who were ≥40 years of age and registered between January 2013 and December 2018 at the Tata Memorial Centre, Mumbai. Diagnosis of ES was confirmed by a specialty pathologist and was based on the essential criteria recommended by the current WHO classification, i.e., round cell morphology and membranous MIC2/CD99 expression [19]. Certain cases with equivocal features were confirmed by molecular testing (EWSR1 gene rearrangement by FISH). The geriatric age group was defined as >60 years as per the prevalent norms. Before the treatment started, all patients were subjected to staging workup. This comprised X-rays and magnetic resonance imaging of the affected area including the proximal and distal joint, as well as an entire-body Positron Emission Tomography-Computed Tomography (PET-CT) scan. Complete hemogram, serum parameters defining renal and liver functions, echocardiography and Diethylenetriaminepentaacetate scan (in selected cases) were carried out to assess optimal organ functioning. At the physician’s discernment, nutritionist advice was sought and deficiencies were corrected at baseline and periodically.

Each patient’s treatment intent and course of therapy was decided in a multidisciplinary Tumour Board (MDT) prior to treatment and at times (especially in recurrent/metastatic/doubtful lesions) after the response to induction chemotherapy (ICT).

**Figure 1.**
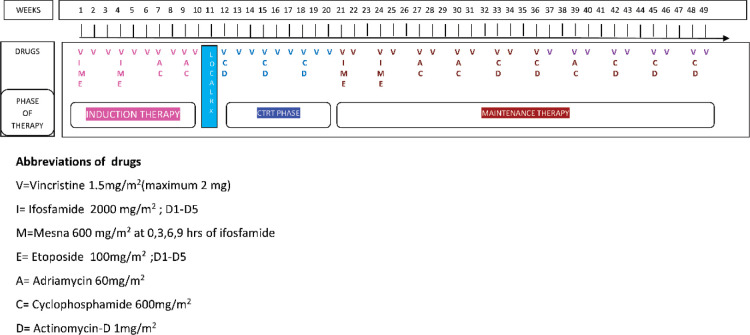
EFT-2001 protocol.

For staging purposes, we used American Joint Committee on Cancer (AJCC) 7th edition of staging [20]. Extra-skeletal tumours with regional lymph node involvement were non-metastatic as per AJCC 7th edition and were managed as stage III as per the institutional practice. The standard in-house Ewing family of tumours (EFT)-2001 regimen [21, 22], Children’s Oncology Group (COG) protocol [23], or standard in-house EFT salvage protocol [24], as well as local therapy, which could be surgery, irradiation, or both, were used for curative purpose. Figure 1 shows the in-house standard EFT-2001 chemotherapy treatment with prophylactic granulocyte colony-stimulating factor. The standard COG protocol was also used to treat a few non-extremity ES patients. The National Cancer Institute Common Terminology Criteria for Adverse Events version 4.03 was used to record chemotherapy-related toxicities until November 2017, after which version 5.0 was used [25, 26]. Chemotherapy dose adoptions were made in accordance with the tolerance and the treating physician’s clinical acumen. After ICT, patients were evaluated for local treatment, and the modality of local therapy was mostly determined by the disease extent and clinico-radiologic response. Options in local therapy were limb salvage or amputation surgery for extremity tumours, wide local excision for non-extremity tumours and/or radiation therapy (RT). Huvos’ necrosis grading was used to ordain the histological response wherein poor response is defined as <90% tumour necrosis [27]. After discussion in our MDT board, patients with positive margins, considerable tumour burden at the time of presentation, contamination of the tumour by prior biopsy or other means and poor necrosis were planned for adjuvant RT. Another response evaluation PET-CT scan was performed 8–12 weeks after definitive RT.

Palliative intent therapy included chemotherapy, oral metronomic chemotherapy (OMCT) [28, 29], surgery/radiation or best supportive care. OMCT comprised a combination of cyclophosphamide (50 mg once a day from D1–D21 every 28 days), etoposide (50 mg once a day D1–D21 every 28 days) and tamoxifen (20 mg twice daily). Data for follow-up was obtained from electronic medical records and updated by telephonic follow-up. The good clinical practice guidelines were followed and the Institute review board granted approval.

### Statistics

The study was carried out using the Statistical Package for the Social Sciences (SPSS) software version 24 (SPSS, Chicago, IL). For continuous variables, the median and interquartile range (IQR) were used, whereas for categorical variables frequency and percent were used. Event-free survival (EFS) was calculated from the date of diagnosis until the first event, which may be progression, relapse, second malignancy or death, whichever came first. The date of diagnosis to the date of death (due to any cause) in dead patients or the date of the last follow-up in alive patients constituted the overall survival (OS). The data cut-off date was 2 February 2021. In the case of recurrent tumours, the event analysis was carried out from the date of relapse to the following event. Age, gender, comorbidities, maximum tumour size, local therapy (surgery versus RT versus both), and other host, tumour, and treatment-related variables were analysed for correlation with survival. Multivariate analysis was used to confirm independent predictability of the factors found significant on univariate analysis.

## Results

### Characteristics of the patients and the tumours

During the 5 years from 2013 to 2018, 1,169 ES patients were registered at our centre. Of these, 66 patients with age ≥40 years were included in the study (Table 1). Out of these 66, 34 (51.5%) were denovo non-metastatic, while 32 (48.5%) were either recurrent (9, 13.6%), metastatic (22, 33.3%) or doubtful metastatic (1, 1.5%). Only two patients had bone marrow involvement at baseline as per fluorodeoxyglucose (FDG) PET-CT. Six patients (9.1%) were in the geriatric age group. Comorbidity was observed in 46.9%, including hypertension in 14 (21.2%), diabetes in 11 (16.7%) and deep vein thrombosis (DVT) or pulmonary embolism in 3 patients (4.5%). The extra-skeletal primary was more common than skeletal primary (63.6% versus 36.4%). The extra-skeletal sites of disease included chest wall, mediastinum, abdominal soft tissue, paravertebral region, perineal region and uterine cervix and vulval soft tissue mass, renal mass, thigh, upper and lower limb soft tissue mass. The commonest primary site was extremities (36.4%) and the sub-site was thigh (10.6%). The median diameter of tumour was 9 (IQR 5.9–13.1) cm. In 57% and 30.5% of cases, serum lactate dehydrogenase (LDH) and serum alkaline phosphatase (SAP) were raised at baseline. Anaemia and hypoalbuminemia were found in 66.7% and 15.8% of the participants, respectively.

### Treatment parameters

**Figure 2.**
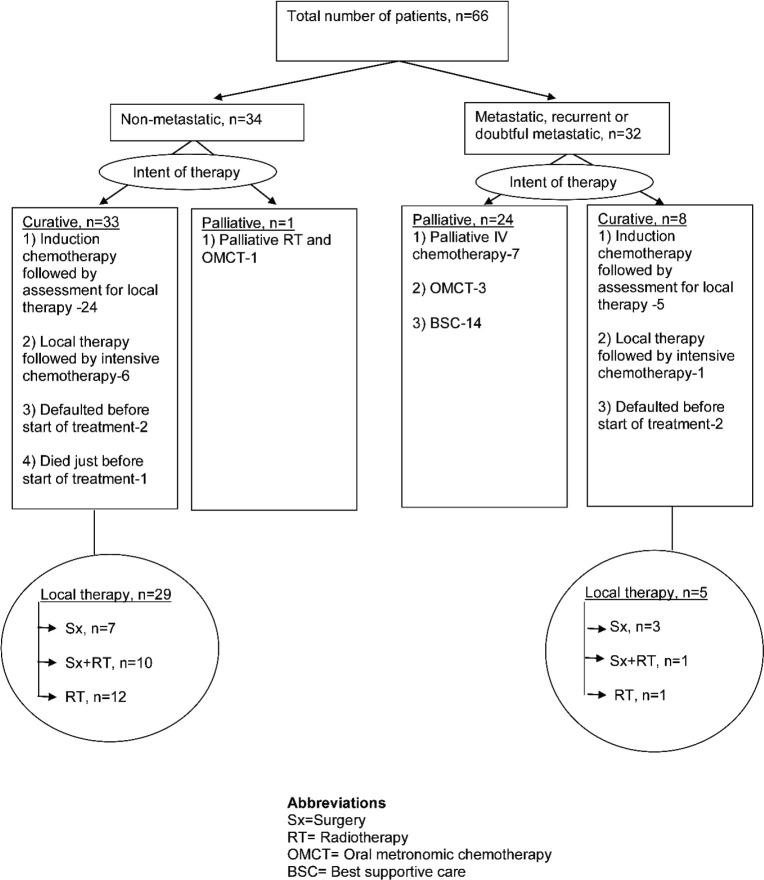
Treatment parameters of Ewing sarcoma patients.

As per the MDT decision, treatment intent was curative for 41 (62.1%) patients, while it was palliative for the remaining 25 cases and is summarised in Figure 2. None of these patients underwent transplant. Of six geriatric patients, four (66.7%) received EFT-2001 chemotherapy (two each with curative and palliative intent) while the remaining two (33.35%) were offered best supportive care.

Non-metastatic cohort (*n* = 34): In this cohort, two defaulted before starting any treatment, and one died of a non-cancerous cause. Hence, only 31 (91.2%) received oncologic treatment. Curative intent local therapy was delivered in 29 (85.3%), which included surgery in 7 (20.6%), surgery and RT in 10 (29.4%), and definitive RT in 12 (35.3%) cases. One patient was planned for palliative intent RT, and one patient defaulted after starting induction therapy. Post resection, good histological necrosis (≥90%) was noted in 57.1%, and three had positive surgical margins. Patients with positive margins received higher doses of RT. Response assessment after 8–12 weeks of completion of definitive radiation was recorded in ten patients, and it showed complete metabolic response in six (60%), partial response in three (30%) and progressive disease in one (10%). Thirty patients received intensive chemotherapy protocols. ICT was delivered in 24 while 6 underwent surgery first. Among patients who underwent surgery first, four were operated at peripheral centres before presentation to our institute, and two underwent surgery at our institute considering pre-operative diagnoses of renal cell carcinoma and angiosarcoma. Diagnosis of ES was established post-surgery in the latter two cases. The intravenous (IV) chemotherapy regimen included EFT-2001 in 27, COG in 2 and modified EFT-2001 protocol in 1. One patient with poor performance status (PS), history of ischemic heart disease and disease progression in the 4 months gap between local therapy and the start of systemic therapy was offered first-line OMCT. Of patients who received any tumour-directed therapy [31], 20 (64.5%) completed the intended therapy.

Metastatic cohort (denovo/recurrent or doubtful), (*n* = 32): Among these, curative-intent local therapy was delivered to five patients. Of these, four had ICT followed by local therapy, including resection in three and definitive RT in one. The other patient underwent upfront surgery along with intraoperative brachytherapy. Limb salvage was done in two of four surgically treated patients. Post resection, good histological necrosis (≥90%) was noted in 66.7%, and none had positive surgical margins. Three patients received lung bath (external beam radiation to both lungs) for lung metastasis. Curative chemotherapy regimen included EFT-2001 in five cases, EFT salvage protocol in one case, while palliative intent chemotherapy, EFT-2001 was given in five cases, and COG protocol in two cases. Of 13 patients receiving intravenous chemotherapy (both curative and palliative), only 5 patients completed the intended treatment. The reasons for non-compliance included severe chemo toxicity in four (including toxic death in three and grade 4 toxicity in one) and logistics in other four. Fourteen patients were declared best supportive care upfront, referred to the palliative care department, and three received OMCT.

### Adverse events

#### Non-metastatic cohort (n = 34)

Total 31 patients received systemic therapy, including IV chemotherapy in 30 and OMCT in 1. Only patients who received IV chemotherapy experienced grade ≥3 adverse events. Notable grade ≥3 haematological chemotherapy related toxicities included febrile neutropenia (FN) in 19 (61.3%), anaemia in 18 (58.1%) and thrombocytopenia in 14 (45.2%). The non-haematological toxicities were chemotherapy-induced nausea/vomiting in two (6.5%), peripheral neuropathy in eight (25.8%), dyselectrolytemia in eight (25.8%), central nervous system (CNS) toxicity in one (3.2%), renal toxicity in two (6.5%), gastrointestinal toxicity in five (16.1%). Twenty (64.5%) patients required dose modification, omission or substitution during chemotherapy. Vincristine was the drug most commonly modified/omitted due to constipation in 2 (6.5%) and peripheral neuropathy in 12 (38.7%) cases. No patient died due to chemotherapy-related toxicity.

#### Metastatic cohort (denovo/recurrent or doubtful) (n = 32)

Of 32 patients, 13 received IV chemotherapy and 3 received OMCT. Hence, total 16 patients were analysed for toxicity data. No significant grade 3 or higher toxicities were observed with OMCT. Notable grade ≥3 toxicities were anaemia in six (37.5%), FN in six (37.5%) and thrombocytopenia in four (25.0%), peripheral neuropathy in two (12.5%), dyselectrolytemia in one (6.25%) and renal toxicity in one (6.25%). Chemotherapy toxicity led to death in three patients. The contributory factors for these deaths included FN and subsequent septicaemia during the induction phase of COG in one, EFT-2001 in another patient in the geriatric age group, while it was acute kidney injury in the induction phase of EFT-2001 in one patient. Dose modification was carried out in other three patients. Vincristine was again the drug most commonly modified/omitted due to ≥grade 2 peripheral neuropathy in nine (56.25%) cases.

### Survival

**Figure 3.**
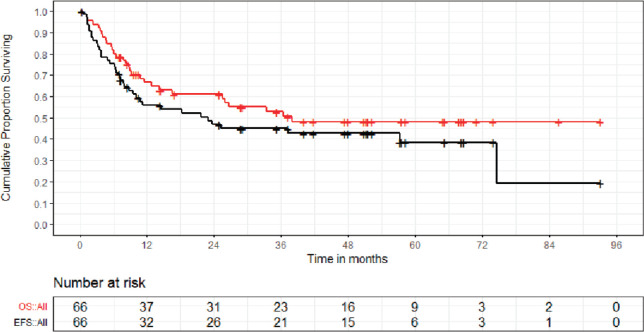
OS and EFS of whole cohort of Ewing sarcoma patients.

**Supplementary Figure 1.**
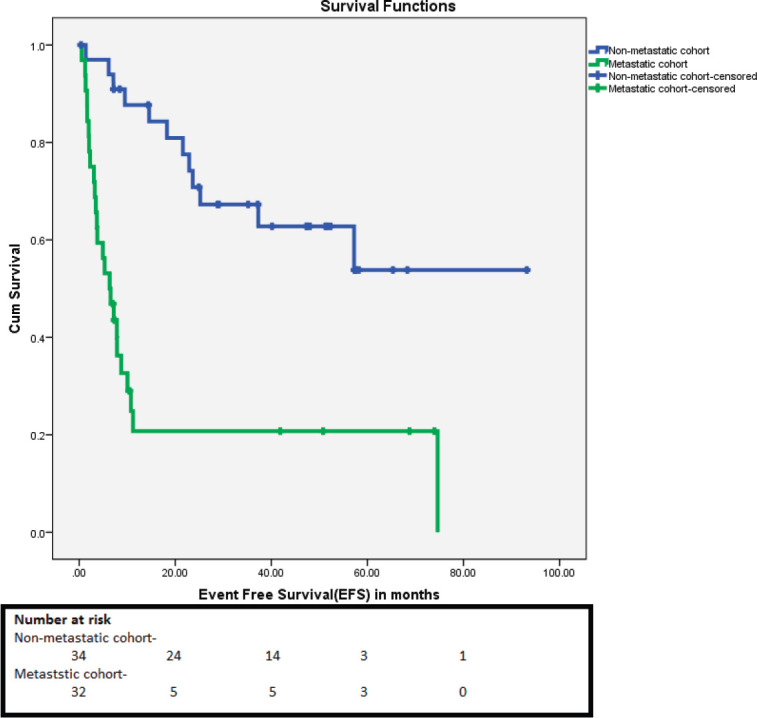
Event free survival (EFS) for non-metastatic and metastatic cohort of Ewing sarcoma patients.

**Supplementary Figure 2.**
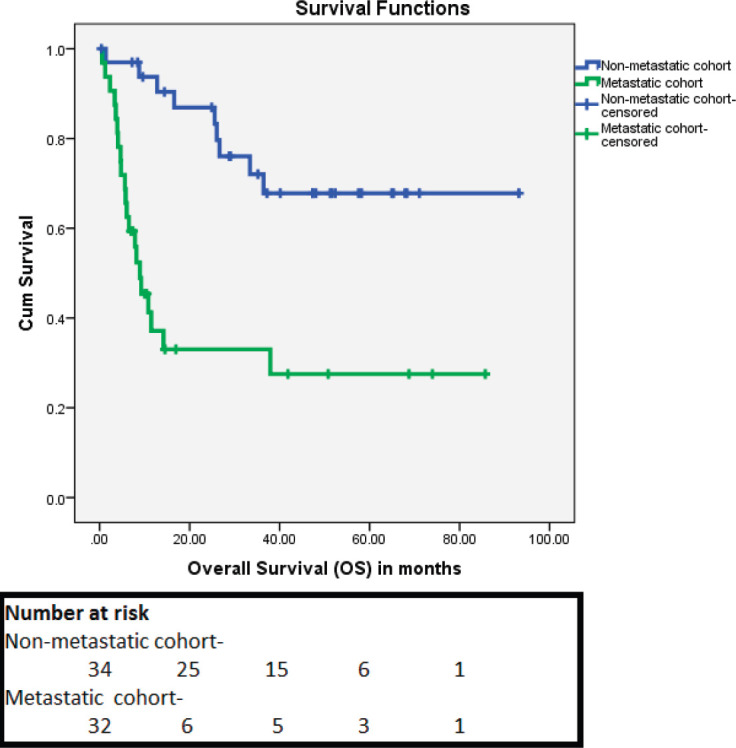
Overall survival (OS) for non-metastatic and metastatic cohort of Ewing sarcoma patients.

At a median follow-up of 48.0 (IQR 24.9 -65.3) months, the median EFS and OS for the entire group were 22.9 (95% CI = 9.2 -36.3) months and 37.9 [95% CI = 14.2- not applicable (NA)] months, respectively. Projected 5 year EFS and OS were 38.4% (95% CI = 24.5–52.3) and 48.1% (95% CI = 34.6–61.6), respectively (Figure 3). The median EFS and OS for non-metastatic cohort were not reached with a projected 5 years EFS and OS of 53.8% and 67.8%, respectively. For metastatic cohort, the median EFS and OS were 6.3 and 8.9 months, respectively. Estimated 5 years EFS and OS for metastatic cohort were 20.7% and 27.5%, respectively. The survival and patterns of failure of the metastatic and non-metastatic cohort have been detailed in Table 2. Also, Supplementary Figures 1 and 2 depict the EFS and OS of two groups, i.e., non-metastatic and metastatic cohort, respectively.

### Prognostic indicators

Metastasis at presentation and a poor Eastern Cooperative Oncology Group Performance Status (ECOG PS) were unfavourable prognostic variables in both EFS and OS. Additionally, raised LDH, maximum tumour size >8 cm, and palliative intent treatment predicted worse EFS. On the contrary, extra-skeletal primary and female gender were indicators of worse OS. The significant independent prognostic factors are summarised in Table 3.

## Discussion

ES in older individuals above the age of 40 is uncommon and poses many challenges in management. They come under the exclusion criteria in most of the randomized trials, and there is a lack of high-quality guidelines, especially as they have various comorbidities which need to be considered while planning the treatment. During our study period, 5.6% of the total ES patients were ≥40 years of age. Analysis of the SEER database from 1973–2008 revealed it to be 13.8% of all ES patients [30]. Approximately 10% were geriatric patients (over 60 years) in our study, and only a few case reports share their experience treating this rare population [31–35]. Extremities were the primary tumour location in 36.4% of cases, and this similar to other studies [16, 36]. The majority of the patients (63.6%) had extra-skeletal primary tumours. In a multicentric study of ES Family of Tumours in patients older than 50 years, over a span of 12 years, Rochefort *et al* [16] identified 77 older patients and 76.6% of these had primary in extra-skeletal sites. A similar observation was noted in another retrospective study of the Surveillance Epidemiology and End Results database from 1973 to 2008 by Karski *et al* [30]. So, the higher number of extra-skeletal primary (63.6%) observed in our study is in sync with other published studies and rather an interesting finding. It contrasts ES in younger patients who have skeletal primaries in the majority of the cases [37, 38]. More than 2/3rd of metastatic, recurrent or doubtful metastatic patients presented with a tumour size ≥8 cm. A similar observation was made by another study reporting larger mean tumour size in metastatic patients [39]. A total 43 of 66 patients (65.2%) received intensive chemotherapy in our study, including 30 (45.5%) in the non-metastatic cohort and the rest 13 (19.7%) from the metastatic cohort, which was similar to the SEER data analysis of elderly ES patients [36]. Of the total 32 patients in the metastatic cohort, 14 patients had widespread metastatic disease. Widely disseminated disease at presentation, upfront marrow, liver, brain and multiple bone metastasis (>3 bone metastases) may be considered for palliative intent systemic therapy; however, the outcomes are dismal. These patients may also be considered for only best supportive care, especially in resource-limited settings [40]. For metastatic ES patients, the Euro-EWING 99 trial [41] had utilised a formal scoring system to decide on the intent of treatment. The Euro-EWING 99 trial had patients who were younger than 50 years. Also, the median age was 16.2 years (range, 0.4 to 49 years) in the Euro-EWING 99 trial while it was 46 (range 40–61) years in the non-metastatic cohort and 45 (range 40–66) years in the metastatic cohort. Moreover, patients with isolated lung metastases were not included in the Euro-EWING 99 trial. Since this is the real world data of older patients at a low and middle income countries (LMIC) setting, no formal scoring system was utilized. However, the decision of treatment was taken in a MDT board in conjunction with the patient and patients’ relative(s) after proper counselling as per the standard institutional practice.

Haematological toxicities were common along with peripheral neuropathy, which was contributed mainly by the use of vincristine. We had three chemotherapy toxicity-related deaths in the metastatic setting. This risk of toxicity highlights the hurdles faced by the adult ES population as a significant proportion of our patients had comorbidities, which compromised health reserves and were unable to tolerate the dose intense chemotherapy regimens. These patients are also more prone to peripheral neuropathy as many are diabetic and more susceptible to neurotoxic chemotherapeutic agents [42]. Dose modification, omission or substitution during chemotherapy was carried out for approximately 2/3rd (64.5%) in the non-metastatic cohort and 1/3rd (37.5%) in the metastatic cohort. In this cohort vincristine was the chemotherapeutic drug which was most commonly modified/omitted. In our earlier study of adolescent-adult (AA) non-metastatic ES patients [43], we have noted that the permanent dose modifications for the entire regimen were required predominantly for haematological toxicities in 34 (14.5%) cases. There were transient or permanent alterations in vincristine doses and/or frequency of administration in additional 83 (35.3%) cases due to peripheral neuropathy (grade ≥2). Few studies also show that delivery of chemotherapy to elderly patients remains a challenge compared to younger patients [44, 45] in view of toxicity and lesser dose intensity achieved in older patients [38, 43, 46]. Cardiac toxicity comparison of older patients with paediatric and AA cohort reveals a higher incidence of cardiac toxicity for children [43, 47] as they are more sensitive to anthracyclines [48].

Four of 66 patients (6.1%) received OMCT in our study, which is well tolerated with relatively few side effects. One randomised control trial evaluated the efficacy of OMCT and found it to be no better than placebo in primary paediatric malignant tumours, including ES. However, these patients were heavily pre-treated, and all progressed after at least two lines of chemotherapy [49]. The role of upfront OMCT in adult patients with ES and those who are unfit for intensive chemotherapy is yet to be explored. We have one 42-year-old metastatic ES patient who responded well to OMCT and obtained a complete response [28]. Outcomes of OMCT in this current study could not be compared to other modalities of therapy because of a lesser number of patients in the OMCT group.

Our finding of inferior survival in older adults is in concurrence with many studies [15, 30, 36, 50–52], including our data of non-metastatic AA ES [43]. The worse outcome can be explained by less physiological reserve and more comorbidity in this group leading to more treatment-related toxicity, with even some curative patients not being considered for intensive chemotherapy owing to these reasons. The inferior outcome can also be partly due to probable differences in tumour biology in older patients developing cancer at an advanced age, as proposed by Karski *et al* [30]. But other retrospective studies have shown that outcomes with intensive regimes in adults mirror that of adolescents [13, 17].

Our finding of extra-skeletal tumours as a bad prognostic factor is in contrast with the analysis by COG [53]. However, the COG findings were based on a trial that included individuals ≤40 years age and we cannot rule out the possibility of age-related physiological differences. Similar to our study, worse survival for extra-skeletal tumours in older patients has been reported [17, 54]. Larger tumour size and higher LDH had poorer outcomes as they indicate greater tumour burden and is echoed in other studies as well [16, 39, 55].

Overall, the literature supports intensive chemotherapy for adult patients of ES at the cost of increased toxicity. However, as seen in our study, toxicities are significant and can lead to adverse outcomes. Precision care must be exercised, and patients should be adequately evaluated clinically and biochemically for fitness for multi-agent therapy, and adequate control of comorbidity should be ensured. Older adults should undergo assessments of physical function and reserve so as to differentiate between the ‘fit older-adult’ and the ‘frail older-adult’. Assessment of PS, activities of daily living and comprehensive geriatric assessment can help select the optimal patients for intensive chemotherapy [56]. Even during the course of therapy, prophylaxis with colony-stimulating factors and frequent review to assess toxicity should be carried out. Dose reduction or dose omissions may be helpful to mitigate toxicity and morbidity and ensure optimal outcomes. To avoid negatively impacting the quality of life of these patients, especially those with elderly metastatic or recurrent ES with a median lifespan of a few months, one must be cautious and appropriately consider the risk versus benefit of treatment.

Our study has various limitations, including the fact that it is a single institute study with a smaller number of patients. However, ES per se is a rare disease and among older population almost an ultra-rare presentation, so numbers would be less, still these are important studies to enhance knowledge in such data sparse zones. Despite small numbers, the relative numbers of events were not relatively less, hence, we proceeded with multivariate analysis to identify predictors. Moreover, the toxicity data encompasses higher grades of toxicities and not of all grades. However, these are therapeutically more relevant. Conducting randomised trials in ultra-rare malignancies like elderly ES is difficult and good quality prospective and retrospective studies will continue to enhance our knowledge and understanding of the disease.

## Conclusion

Older adult ES patients with good PS and physiological reserve benefit from multimodality standard of care treatment, which improves outcomes even in resource-constrained settings in LMIC. However, this requires careful patient selection and mandates close monitoring under expert care for optimising management of potential toxicities. The role of upfront OMCT and less aggressive chemotherapy needs to be explored further for patients unfit for intensive chemotherapy regimens.

## Abbreviations

ES: Ewing sarcoma
LMIC: Low and middle income country
EFT-2001: Ewing family of tumours-2001
COG: Children’s oncology group
ICT: Induction chemotherapy
MCT: Maintenance chemotherapy
PS: Performance status
LDH: Lactose dehydrogenase
SAP: Serum alkaline phosphatase
OS: Overall survival
EFS: Event free survival

## Conflicts of interest

The authors declare that they have no known competing financial interests or personal relationships that could have appeared to influence the work reported in this paper.

## Funding

This research did not receive any specific grant from funding agencies in the public, commercial, or not-for-profit sectors.

## Ethics approval

Approval from institutional ethics committee (IEC) was taken and waiver of consent was obtained from IEC.

## Availability of data and material

The data that support the findings of this study are available on request from the corresponding author. The data are not publicly available due to privacy or ethical restrictions.

## Figures and Tables

**Table 1.** Baseline patient and tumour characteristics.

Characteristics	Non-metastatic (*n* = 34) (100%)		Metastatic, recurrent or doubtful metastatic^a^ (*n* = 32) (100%)	
Patient characteristics				
Age in years Median (Range)	46 (40–61)		45 (40–66)	
Gender Male Female	24 (70.6%) 10 (29.6%)		19 (59.4%) 13 (40.6%)	
ECOG PS 0–1 2 >2	22 (64.7%) 4 (11.8%) 8 (23.5%)		18 (56.2%) 6 (18.8%) 8 (25%)	
Comorbidity Yes No	15 (44.1%) 19 (55.9%)		16 (50%) 16 (50%)	
Family history of malignancy Yes No	2 (5.9%) 32 (94.1%)		0 (0%) 32 (100%)	
Tumour characteristics				
6. Tumour location I) Extremity Upper extremity Lower extremity II) Non-extremity Head and neck Pelvis Paravertebral Chest wall Visceral organ Other	10 (29.4%) 3 (8.8%) 7 (20.6%) 24 (70.6%) 4 (11.8%) 4 (11.8%) 6 (17.6%) 2 (5.9%) 2 (5.9%) 6 (17.6%)		14 (43.8%) 2 (6.3%) 12 (37.5%) 18 (56.3%) 0 (0%) 4 (12.5%) 2 (6.3%) 1 (3.1%) 3 (9.4%) 8 (25%)	
Primary tumour Extra-skeletal Skeletal	18 (52.9%) 16 (47.1%)		24 (75%) 8 (25%)	
Maximum tumour size <8 cm ≥8 cm	19 (55.9%) 15 (44.1%)		9 (28.1%) 23 (71.9%)	
Skip lesion Yes No	2 (5.9%) 32 (94.1%)		5 (15.6%) 27 (84.4%)	
10. Regional lymph node involvement Yes No	3 (8.8%) 31 (91.2%)		12 (37.5%) 20 (62.5%)	
11. Metastatic sites Lung only Bone only Sites other than lung or bone	NA NA NA		5 (15.6%) 5 (15.6%) 18 (56.3%)	
12. Number of metastasis 1–5 >5	NA NA		3 25	
13. Intent of treatment Curative Palliative	33 (97.1%) 1 (2.9%)		7 (21.9%) 25 (78.1%)	
Laboratory parameters	Median IQR		Median IQR	
14. Haemoglobin (g/dL)	12.4	11.5–13.8	10.5	9.8–12.7
15. Albumin (g/dL)	4.3	3.9–4.5	3.9	3.4–4.3
16. SAP (U/L)	100	90–142.5	109	84.5–141
17. LDH (U/L)	171	145–310	396	192–1,025

ECOG PS = Eastern Cooperative Oncology Group Performance Status, SAP = Serum alkaline Phosphatase, LDH = Lactate dehydrogenase

a Three patients were recurrent non-metastatic and one patient was doubtful metastatic

**Table 2.** Survival outcomes and patterns of failure.

	Median follow up	Median EFS	Median OS	5 year EFS	5 year OS	Patterns of failure
Non-metastatic cohort (*n* = 34)	48.0 (IQR 29.1–64.9) mths	NR	NR	53.8% (95% CI 31.5–76.1)	67.8% (95% CI 50.2–85.4)	Five distant Four loco-regional Two both distant and loco-regional
Metastatic cohort (denovo/recurrent or doubtful) (*n* = 32)	41.9 (IQR 14.5–68.8) mths	6.3 (95% CI 3.3–9.4) mths	8.9 (95% CI 5.4–12.5) mths	20.7% (95% CI 5.6–35.8)	27.5% (95% CI 9.9–45.1)	14 distant Two loco-regional Six both distant and loco-regional

EFS = Event free survival, OS = Overall survival, IQR = Inter quartile range, CI = Confidence Interval, NR = Not reached, mths = Months

**Table 3.** Significant prognostic factors in multivariate analysis.

Cohort	Factors affecting EFS	Factors affecting OS
Overall cohort	1. Metastatic VS non-metatastatic	1. Metastatic VS non-metatastatic
	(*p* = 0.022; HR = 4.86 (1.26–18.9))	(*p* = 0.002; HR = 5.1 (1.8–14.5))
	2. ECOG PS 0–2 VS >2	2. Extra-skeletal VS skeletal primary
	(*p* = 0.008; HR = 8.8 (1.7–39.1))	(*p* = 0.00; HR = 6.9 (2.4–19.6))
	3. LDH raised VS not raised	3. Female VS male
	(*p* = 0.024; HR = 4.17 (1.25–14.29))	(*p* = 0.016; HR = 3.1 (1.2–7.7))
	4. Maximum tumour size ≥ 8 cm VS <8 cm	4. ECOG PS 0–2 VS >2
	(*p* = 0.017; HR = 3.57 (1.25–10.15))	(*p* = 0.002; HR = 5.1 (1.9–14.2))
	5. Intent of treatment: palliative VS curative	
	(*p* = 0.005; HR = 41.8 (3.1–572.4))	
Non-metastatic	1. LDH raised VS not raised	1. LDH raised VS not raised
	(*p* = 0.007; HR = 0.1 (0.02–0.53) )	(*p* = 0.008; HR = 33.3 (25–333.3))
Metastatic, recurrent or doubtful metastatic	1. Extra-skeletal VS skeletal primary	1. Extra-skeletal VS skeletal primary
	(*p* = 0.004; HR = 7.214 (1.9–27.3))	(*p* = 0.004; HR = 20.6 (2.6–165.3))

VS = Versus, EFS = Event free survival, OS = Overall survival, LDH = Lactate dehydrogenase, ECOG PS = Eastern Cooperative Oncology Group Performance Status
